# Circulating miR-19b and miR-181b are potential biomarkers for diabetic cardiomyopathy

**DOI:** 10.1038/s41598-017-13875-2

**Published:** 2017-10-18

**Authors:** Camila Uribe Copier, Luis León, Mauricio Fernández, David Contador, Sebastián D. Calligaris

**Affiliations:** 1 Centro de Medicina Regenerativa, Facultad de Medicina, Clínica Alemana Universidad del Desarrollo, Av. Las Condes 12.438, Lo Barnechea, Santiago Chile; 2 Instituto de Ciencias Biomédicas, Facultad de Ciencias de la Salud, Universidad Autónoma de Chile, Pedro de Valdivia 425, Providencia, Santiago Chile; 3 Departamento de Cardiología, Clínica Alemana de Santiago - Universidad del Desarrollo, Vitacura 5951, Vitacura, Santiago Chile

## Abstract

Diabetic cardiomyopathy is characterized by metabolic changes in the myocardium that promote a slow and silent dysfunction of muscle fibers, leading to myocardium remodelling and heart failure, independently of the presence of coronary artery diseases or hypertension. At present, no imaging methods allow an early diagnosis of this disease. Circulating miRNAs in plasma have been proposed as biomarkers in the prognosis of several cardiac diseases. This study aimed to determine whether circulating miRNAs could be potential biomarkers of diabetic cardiomyopathy. Mice that were fed with a high fat diet for 16 months, showed metabolic syndrome manifestations, cardiac hypertrophy (without hypertension) and a progressive cardiac function decline. At 16 months, when maximal degree of cardiac dysfunction was observed, 15 miRNAs from a miRNA microarray screening in myocardium were selected. Then, selected miRNAs expression in myocardium (at 4 and 16 months) and plasma (at 4, 12 and 16 months) were measured by RT-qPCR. Circulating miR-19b-3p and miR-181b-5p levels were associated with myocardium levels during the development of diabetic cardiomyopathy (in terms of cardiac dysfunction), suggesting that these miRNAs could be suitable biomarkers of this disease in asymptomatic diabetic patients.

## Introduction

Diabetes Mellitus (DM) has shown an increasing prevalence since 1980. In 2014, it was estimated that 422 million adults lived with DM and approximately 4.9 million people died from complications related to diabetes, and positioned cardiovascular disease as the major cause of death^1^.

Diabetic patients could have an asymptomatic cardiac function alteration without coronary arterial diseases, hypertension, or any congenital cardiac disease. This manifestation was defined as diabetic cardiomyopathy in 1972, and its implications in the integral treatment of cardiac disease related to DM in patients have aroused increasing interest in the scientific and clinical community^2,3^. During the latency period of the diabetic cardiomyopathy, alterations of the energetic metabolic and myocardial structure lead to a diastolic dysfunction, which progresses to concentric ventricular hypertrophy and a reduction of its contractile reserve, eventually reaching systolic dysfunction^4^.

In a population-based study performed in Olmsted County (USA), 1.1% of the community met criteria for diabetic cardiomyopathy, increasing to 16,9% in asymptomatic diabetic patients, of which 54.4% had diastolic dysfunction^5^. In another study in Bruderholz (Switzerland), 58% of diabetic patients, without previous evidence of structural heart diseases, were diagnosed with diabetic cardiomyopathy^6^.

Currently, therapy for diabetic cardiomyopathy is based on strict glycemic control, changes in lifestyle favoring exercise, balanced calorie intake and administration of beta-blockers and angiotensin converting enzyme inhibitors. Several observational studies show that the effectiveness of these treatments increase significantly when diabetic cardiomyopathy is detected in its early stages^7^.

The lack of specific characteristics makes the detection of diabetic cardiomyopathy a challenge in clinical practice. The diagnosis in asymptomatic diabetic patients without hypertension and coronary diseases is based on the detection of cardiac hypertrophy and diastolic dysfunction by echocardiography^8^.

Currently no blood or imaging test is available for diagnosis prior to irreversible heart remodeling; however, circulating long-non-coding RNA or microRNAs (miRNAs) have been proposed as new type of biomarkers for cardiovascular diseases, mainly because they are involved in epigenetics mechanisms of cardiomyopathies^9–11^. MiRNAs are small non-coding RNA molecules that function as post–transcriptional regulators, decreasing expression of target genes and regulating multiple cellular pathways. A key attribute that support their use is their stability in plasma, because they are within exosomes and high density lipoproteins (HDL) and can form complex soluble proteins.

One approach to discover novel miRNAs as biomarkers, is the use of animal models to correlate their gene expression patterns in the damaged-tissue with their levels in biofluids. This strategy has the advantage of strongly associating a circulating miRNA signature with a specific organ state in a multiple DM-related complications scenario^12^. In the present study, we used a diabetic cardiomyopathy mice model, induced by a high-fat diet, to correlate the miRNA relative abundance in plasma with their expression in myocardium and cardiac function decline during the development of diabetic cardiomyopathy secondary to obesity. We identified two miRNAs whose relative abundance in plasma is reduced before cardiac dysfunctions appear; thus, we propose them as potential biomarkers for diabetic cardiomyopathy.

## Results

### Diabetic cardiomyopathy animal model characteristics

**Figure 1:**
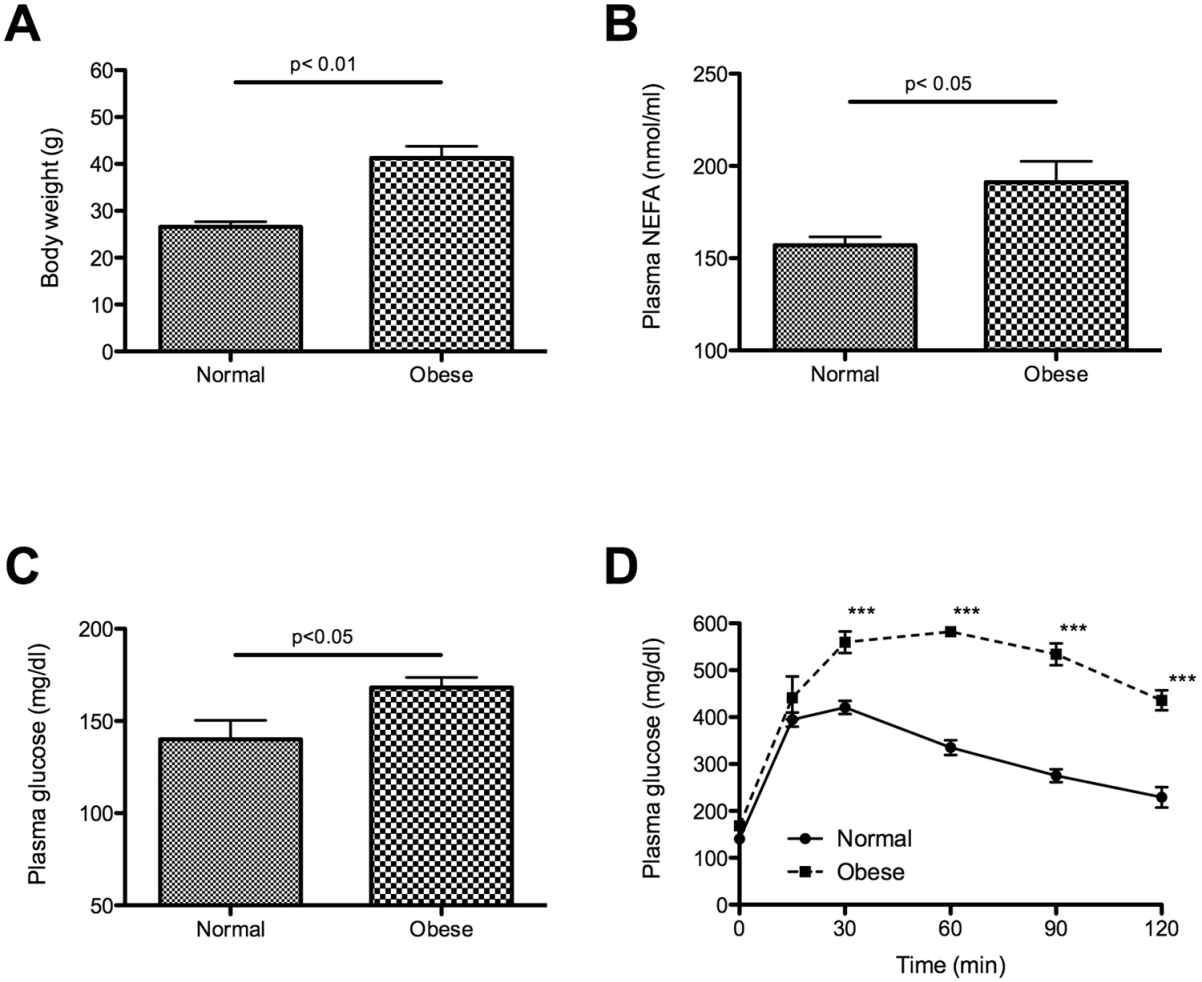
Characteristics of the obesity animal model induced by high fat diet. After 4 months of high-fat diet treatment, body weight (**A**), plasma NEFA, glucose (**B** and **C** respectively), and a glucose tolerance test (**D**) were assessed. (n = 6–7) Mean ± SEM, *p < 0.05, **p < 0.01, ***p < 0.001 vs. normal mice (t-Student test and Two-way ANOVA test).

Animals fed with a high-fat diet for 4 months (at the beginning of the study) were overweight (Fig. 1A), and they presented elevated plasma non-ester fatty acid (NEFA) levels (Fig. 1B), hyperglycaemia (Fig. 1C), and glucose intolerance (Fig. 1D) in comparison with mice fed with a standard diet. All of these features in the high-fat diet mice are characteristics of metabolic syndrome, which remained until the end of the study^13^.

With regard to cardiovascular characteristic of the animal model, mean arterial pressure slightly decreased during the whole dietary treatment in obese mice, without statistical difference between both experimental groups (Fig. 1SA). However, after 4 months of dietary treatment, the obese mice (O) presented a heart mass higher than that of normal mice (N), resulting in cardiac hypertrophy (160 ± 4 mg, O *vs*. 131 ± 3 mg, N; p < 0.001), which remained until the end of the study (Fig. 1SC). Consistent with these results, the cross-sectional area of cardiomyocyte was higher in obese myocardium in comparison to normal myocardium (Fig. 1SD). Interestingly, normal mice underwent an age-related cardiac hypertrophy (Fig. 1SC). In addition, left ventricular end-diastolic pressure (LVEDP) was also higher in obese mice, which is a functional indicator of cardiac remodelling (Fig. 1SB). The obese mice had cardiac hypertrophy despite their normotensive state, which is a characteristic of diabetic cardiomyopathy.

**Figure 2:**
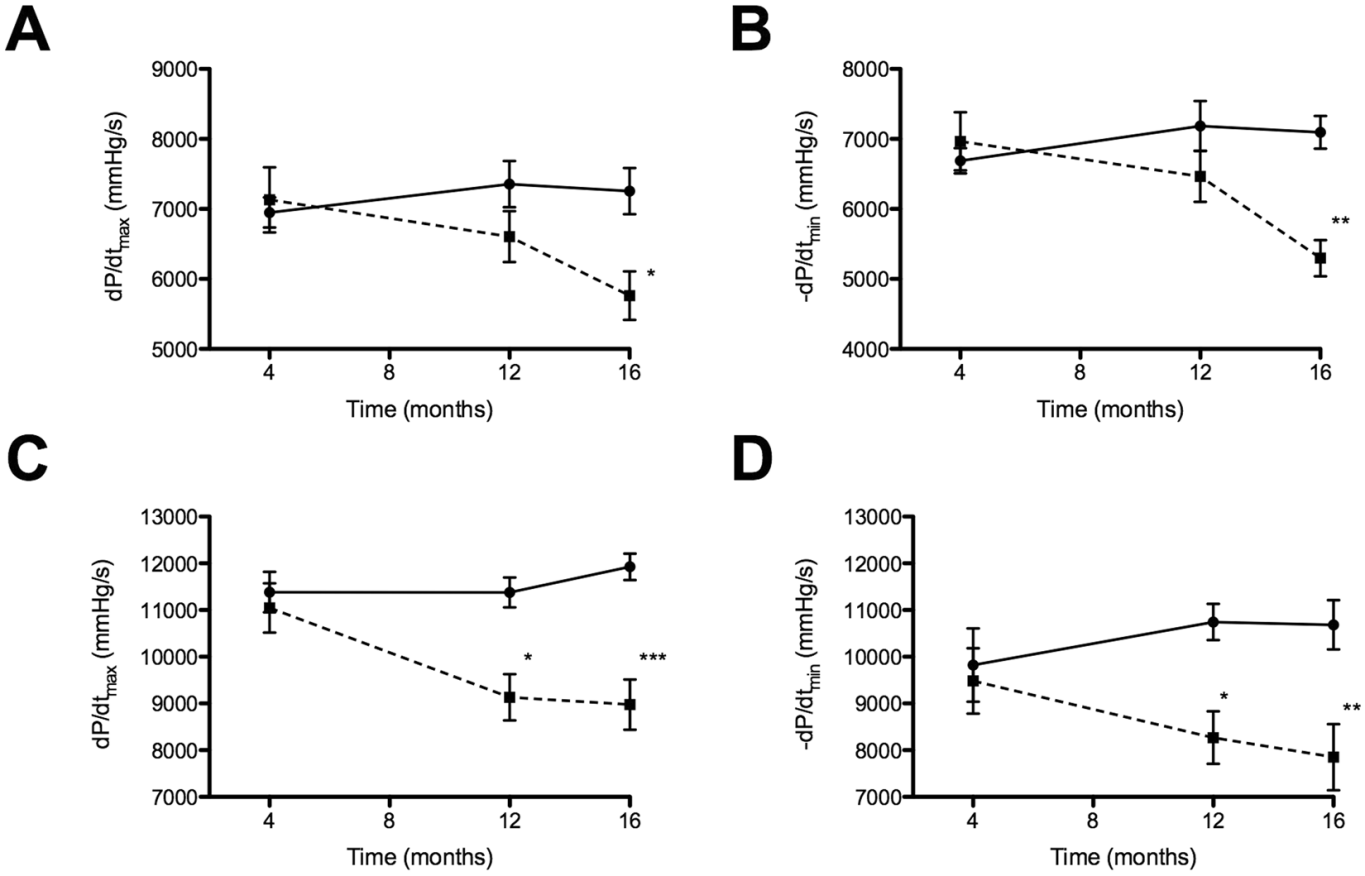
Cardiac function under basal and stress conditions. Cardiac catheterization under basal (**A**,**B**) and stress conditions by dobutamine (**C**,**D**). In the cardiac stress test, max-peak of stimulation with dobutamine were considered to obtained *d*P/*d*t_max_ and *d*P/*d*t_min_. Solid and dotted lines indicate were used to indicate normal and obese mice respectively. (n = 7). Mean ± SEM, *p < 0.05, **p < 0.01, ***p < 0.001 vs. normal mice (Two-way ANOVA test).

Regarding cardiac function at basal condition, high fat diet feeding induced systolic dysfunction (*d*P/*d*t_max_: 5768 ± 341 mmHg, O *vs*. 7255 ± 330 mmHg, N; p < 0.05) and diastolic dysfunction (*d*P/*d*t_min_: −55297 ± 258 mmHg, O *vs*. −7117 ± 243 mmHg, N; p < 0.01) in obese mice only at 16 months (Fig. 2A and B respectively). However, when obese mice were exposed to a cardiac stress test (with dobutamine), systolic and diastolic dysfunctions were observed at 12 months ((*d*P/*d*t_max_: 8975 ± 538 mmHg, O *vs*. 11925 ± 281 mmHg, N; p < 0.05 and *d*P/*d*t_min_: −8270 ± 561 mmHg, O *vs*. −10744 ± 387 mmHg, N; p < 0.05), indicating that the performance of an obese heart is altered before it manifests at basal condition (Fig. 2C y 2D respectively). According to these results, three endpoints in the progression of cardiac dysfunction were defined in order to study search miRNAs useful for biomarker applications.

### MiRNA differentially expressed in myocardium during diabetic cardiomyopathy progression

**Figure 3:**
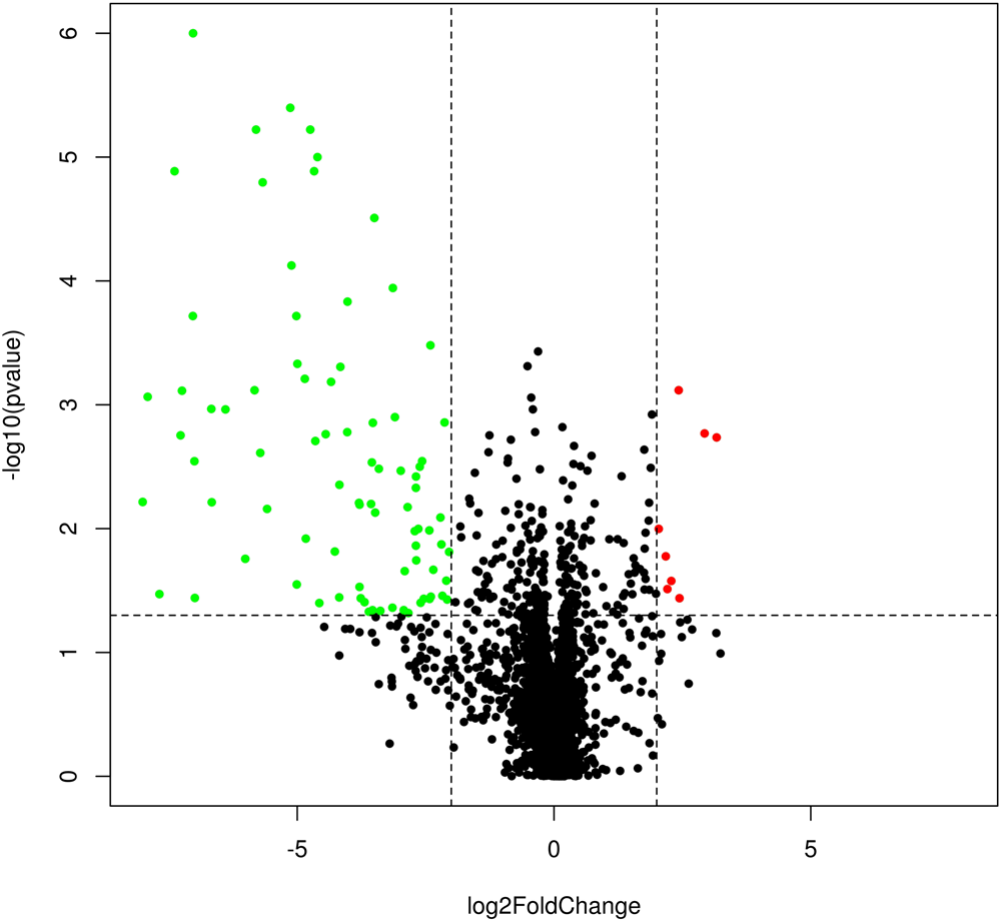
Volcano plot of microRNA microarray data of myocardium from obese and normal mice at 16 months. The y-axis values show the negative logarithm base 10 of the P-value. The x-axis is shown as the log 2 differences in estimated relative expression values. The dotted horizontal line on the plot represents the α-level used for this analysis (0.05). Vertical dotted lines represent the threshold for the log 2-fold change (equivalent to a 2-fold change). Thus, the green and red dots correspond to miRNAs that show a significant 4-fold or higher in expression between obese and normal mice (n = 3).

MiRNA expression profile was comprehensively analysed in myocardium of obese mice at 16 months (maximal degree of cardiac dysfunction) by using Affymetrix miRNA microarray and it was compared with the profile in myocardium of normal mice. The Volcano diagram shows a marked difference in diverse miRNA expression levels (Fig. 3).

To select the miRNA candidates for RT-qPCR validation, three approaches were considered: (1) statistical criteria: 19 miRNAs were differentially expressed with an FDR of 0.1 and Fold Change (FC) > 2 among 3163 miRNA analysed (1908 mature miRNA and 1255 pre-miRNA), see Supplemental Data, Table 1; (2) experimental validated target match: we compared a list of miRNA targets experimentally validated with our list of miRNAs obtained by microarray, finding 17 miRNAs that met this requirement; and (3) biological activity criteria by literature mining. For further details of procedures, see the Methods section.

**Table 1:** Comparison of MiRNAs gene expression in myocardium from our study respect to previously published data, considering their roles in signalling pathways related to diabetic cardiomyopathy.

microRNAs	Relative expression (vs. normal mice)	Biological effect(s) of its regulation previously reported/Signaling pathways and targets involved	Experimental model/tissue or cell types	References
Let-7f	↓	Alteration of glucose homeostasis and insulin sensitivity/INSR	C57BL/6 mice feed with high-fat diet (7 weeks)/liver, muscle and pancreas	20
miR-10a	↓	Increase of hyperlipidemic-induced inflammatory response/Rho kinase and connexin43	Sprague-Dawley rats feed with high-fat diet + streptozotocin /	21
miR-322	↓	Inhibition of the insulin pathway/INRS, IGF1R and cyclin D	C57BL/6 mice feed with high-fat diet (10 weeks)/heart	22
miR-19b	↓	Inhibition of cell survival pathway	H9C2 cardiomyocytes culture exposed to H_2_O_2_ and neonate rat cardiac fibroblast	23
		Inhibition of fibrogenesis pathway/TGF-β RII	Human cardiac fibroblast cells	35
miR-25	↓	Activation of cell apoptosis related to oxidative stress pathways/MCU	H9C2 cardiomyocytes culture exposed to doxorubicin	28
miR-199a	↓	Activation of hypoxic response to avoid cell apoptosis/Hif-1α and Sirt1	Neonatal rat cardiomyocytes	27
miR-30e	↓	Activation of cell apoptosis/Beclin-1	H9C2 cardiomyocytes culture exposed to doxorubicin	24
miR-140	↓	Activation cell apoptosis/Mitofusin I	Neonatal rat cardiomyocytes exposed to H_2_O_2_	26
miR-181a/b	↓	Reduction of basal mitochondrial respiration/Inhibition of PI3K signaling by PTEN increase	Sponge-H9c2 cardiomyocytes culture	29
miR-499	↓	Activation cell apoptosis/Pdcd4, Pacs2 and Dyrk2	H9C2 cardiomyocytes culture exposed to H_2_O_2_	25
miR-146a	↓	Activation of inflammatory genes, fibronectin, MMPs and collagen via Fos - AP1	AC16 cell line (cardiac muscle cells)	30
miR-155	↓	Activation of inflammatory signalling pathways/NF-kB	Diabetes Mellitus type 1 model with Sprague-Dawley rats administrated with streptozotocin	31
miR-3473b	↓	Activation of inflammatory signalling pathways/TRAF3 - NF-kB	Bacterial infection model with Murine macrophages	33
miR-451	↓	It upregulation induces activation of lipotoxicity through suppression of the LKB1/AMPK pathway	C57BL/6 mice feed with high fat diet (20 weeks)/heart; Neonatal rat cardiomyocytes	34

AP-1: Activator protein 1, AMPK: AMP-activated protein kinase, Dyrk2: Dual specificity tyrosine-phosphorylation-regulated kinase 2, INRS: Insulin receptor, IGF1R: Insulin growth factor 1 receptor, LKB1: Liver kinase B1, MCU: Mitocondrial calcium uniporter, MMP: Matrix metalloproteinase, NF-κB: Nuclear factor κB, Pacs2: Phosphofurin acidic cluster sorting protein 2, PTEN: phosphatase and tensin homolog; Pdcd4: programmed cell death 4, Sirt1 Sirtuin 1, TGF-β RII: transforming growth factor beta receptor II, TRAF3: TNF Receptor associated factor 3.

A total of 15 miRNAs (let-7f-5p, miR-10a-5p, miRNA-19b-3p, miR-25-3p, miR30e-5p, miR-140-5p, miR-155-5p, miR-146a-5p, miR-181b-5p, miR-199a-3p, miR-322, miR-451, miR-499-5p, miR-669m-5p and miR-3473b) were selected considering the three approaches.

**Figure 4:**
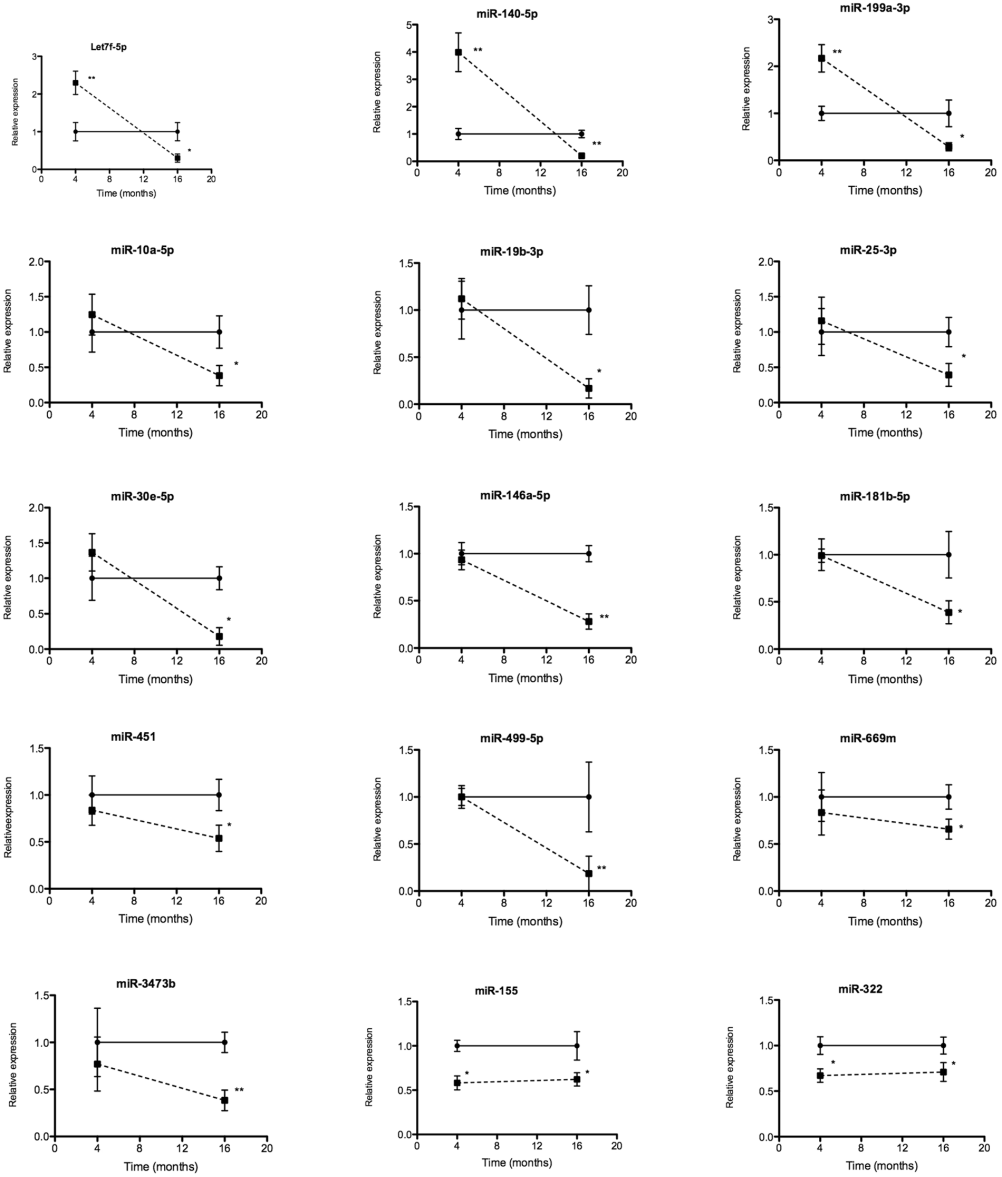
Validation of the differentially expressed miRNAs in myocardium by RT-qPCR. MiRNAs were isolated from myocardium of normal and obese mice after 4 and 16 months of dietary treatment. Solid and dotted lines indicate normal and obese mice respectively. Relative expression of each miRNA was normalized to snoRNA202. (n = 8–9). Mean ± SEM, *p < 0.05, **p < 0.01 vs. normal mice (Two-way ANOVA test).

A summary of validation data of differential expression of miRNAs in the myocardium of obese compared to normal mice at 4 and 16 months is showed in Fig. 4. For normalization procedure, snoRNA202 was used as an endogenous control. At 16 months, all 15 miRNAs were significantly downregulated in heart tissue of obese mice compared to heart tissue of normal mice: let-7f-5p (FC: 3.3), miR-10a-5p (FC: 2.6), miRNA-19b-3p (FC: 5.0), miR-25-3p (FC: 2.6), miR30e-5p (FC: 5.6), miR-140-5p (FC: 5.0), miR-155-5p (FC: 1.7), miR-146a-5p (FC: 4.0), miR-181b-5p (3.0), miR-199a-3p (FC: 3.6), miR-322 (FC: 1.5), miR-451 (FC: 1.9), miR-499-5p (FC: 5.4), miR-669m-5p (FC: 1.7) and miR-3473b (FC: 3.4). At 4 months of high-fat diet feeding, 3 miRNAs were upregulated: let7f-5p (FC: 2.3), miR-140-5p (FC: 3.8) and miR-199-3p (FC: 2.1), but two miRNAs were down regulated in obese mice with respect to normal mice: miR-155 (FC: 1.7) and miR-322 (FC: 1.5). The gene expression of the remaining miRNAs was unchanged within both experimental groups (Fig. 4).

### MiRNA differentially expressed in plasma during diabetic cardiomyopathy progression

**Figure 5:**
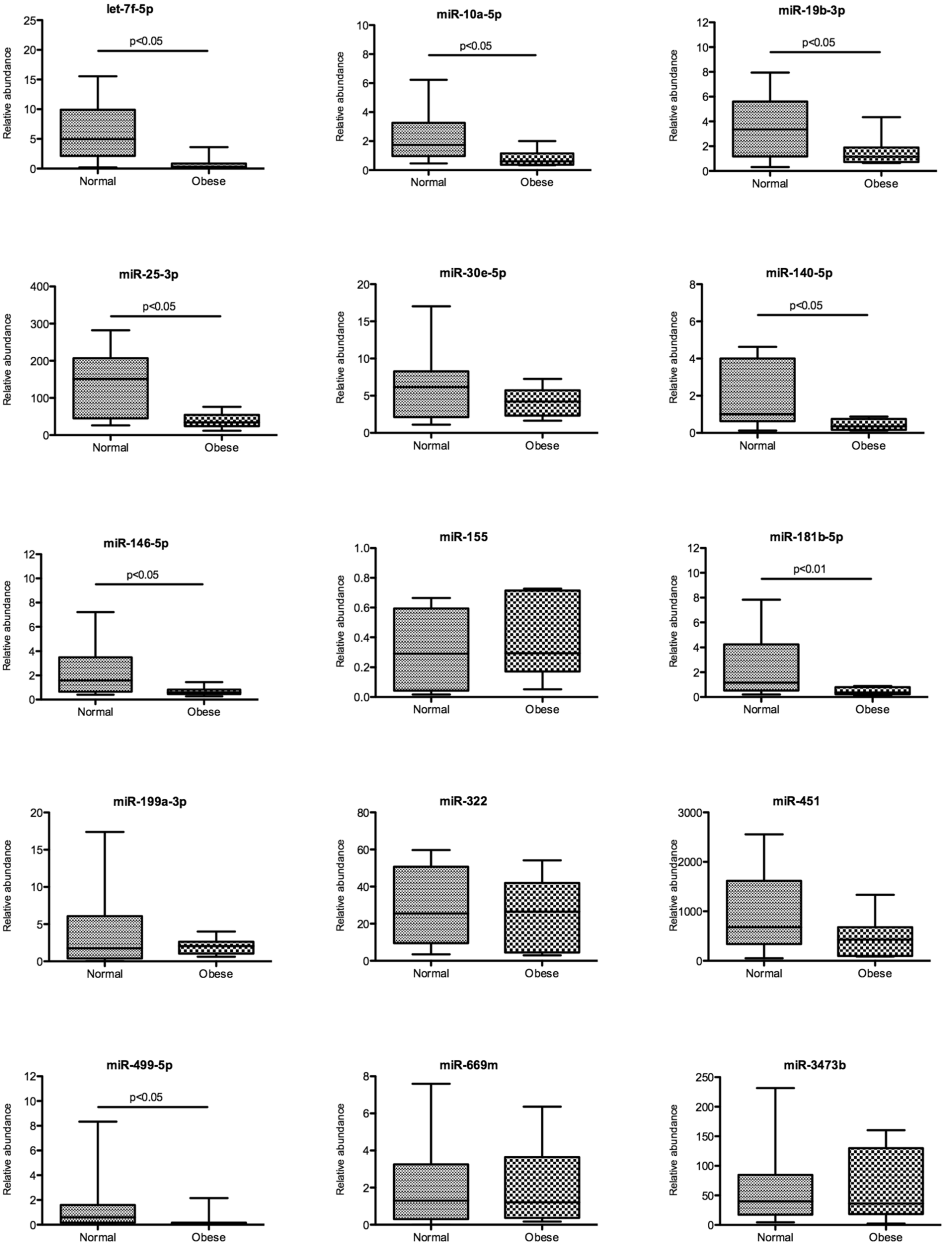
Circulating miRNAs in plasma at 16 months. MiRNAs were assessed at 16 months of dietary treatment. Relative abundance of each miRNA was normalized to cel-miR-39. (n = 8–9). Mean ± SEM, *p < 0.05, **p < 0.01 vs. normal mice (Mann-Whitney test).

**Figure 6:**
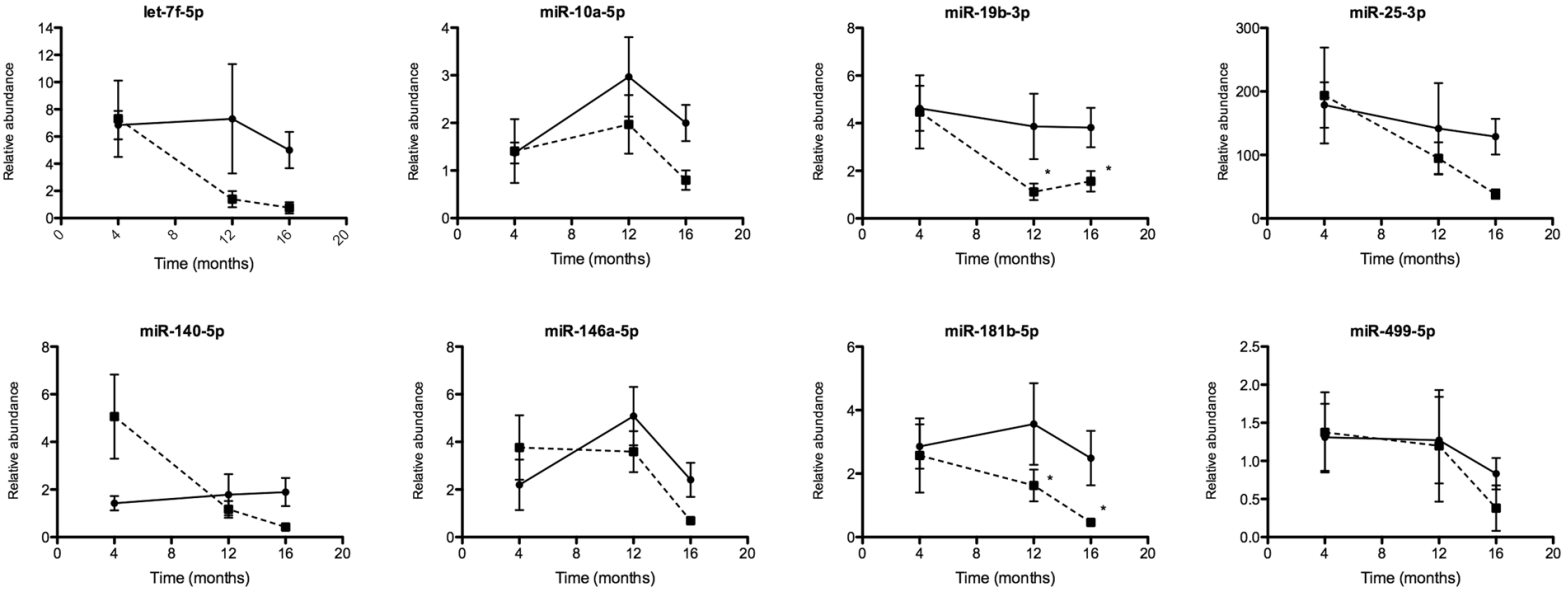
Circulating miRNAs in plasma. MiRNAs were assessed at 4, 12 and 16 months of dietary treatment. Solid and dotted lines indicate normal and obese mice respectively. Relative abundance of each miRNA was normalized to cel-miR-39. (n = 8–9). Mean ± SEM, *p < 0.05 vs. normal mice (Two way ANOVA, Tukey HSD).

In order to select the miRNAs in which plasmatic levels may reflect changes in gene expression in myocardium, we first determined the miRNA relative abundance in plasma of obese and normal mice at 16 months, considering the cel-miR-39 as a spike-in for their miRNA levels normalization (Fig. 5). We found 8 circulating miRNAs that were less abundant in the obese mice than in normal mice, indicating an association between their gene expression in myocardium: let-7f-5p (FC: 5.4), miR-10a-5p (FC: 2.3), miRNA-19b-3p (FC: 2.5), miR-25-3p (FC: 3.4), miR-140-5p (FC: 4.5), miR-146a-5p (FC: 3.3), miR-181b-5p (FC: 5.2) and miR-499-5p (FC: 2.2). Then, we performed a time-course study, measuring these miRNA levels in plasma at 4 and 12 months retrospectively to detect changes in their levels before cardiac dysfunction manifestation. At 12 months we observed a reduction of their abundance in 4 circulating miRNAs: miR-25-3p (FC: 1.5), let-7f-5p (FC: 5.2), miR-181b-5p (FC: 2.0) and miR-19b-3p (FC: 3.4); plasmatic levels were reduced in obese mice during the dietary treatment (Fig. 6). However, only in the last two miRNAs were the differences with respect to normal mice statistically significant (p < 0.04 and p < 0.03 respectively), suggesting that both miRNAs could be useful as biomarkers for diabetic cardiomyopathy. Interestedly, at 4 months, miR-140-5p plasmatic levels were higher in obese mice than in the normal mice (FC: 3.6), according to data obtained from myocardium expression experiments (Fig. 6).

## Discussion

In the present study, we defined three time-points in the development of diabetic cardiomyopathy induced by high-fat diet feeding: an initial stage with metabolic syndrome manifestations and cardiac hypertrophy with normal arterial pressure and cardiac function (4 months); a “subclinical” stage in which cardiac dysfunction is detectable only under stress test condition (12 months); and a last stage in which heart function is decreased at basal condition in obese mice with normal arterial pressure but significant cardiac hypertrophy, which are characteristics of early diabetic cardiomyopathy (16 months)^13^. In previous works, the induction of obesity by high-fat diet did not extend much longer than 5 months, in order to study its effect on cardiac metabolism and function, observing cardiac hypertrophy (as in this study) and a slight cardiac dysfunction in obese mice (C57BL/6 strain) measured by echocardiography^14,15^. On the other hand, according to our results, a recent study described that high-fat diet feeding for 7 months did not alter cardiac function measuring by echocardiography in the same mice strain^16^. An explanation for the differences in cardiac susceptibility to high fat diet between published studies could be due to the use of different substrains of C57BL/6^17,18^.

We found differential expression of several miRNAs in myocardium of mice with diabetic cardiomyopathy compared to that of normal mice. A recent study demonstrated that high-fat diet feeding after five months induced a different miRNA expression pattern in the heart, identifying miR-21a-3p, miR-29c-3p, and miR-195a-3p related to the glucose metabolic pathway^19^. We found the same miRNAs deregulated negatively in obese mice in the microarray study at 16 months, associated to the metabolic syndrome description of hyperglycaemia and glucose intolerance (Table 1S)^13^.

We clearly observed three different miRNA expression patterns in myocardium during the dietary treatment: (1) an initial upregulation of miRNAs expression at 4 months, but their expression was reduced significantly in obese mice during the heart dysfunction development; (2) unchanged miRNAs expression in obese mice at 4 months, with an evident reduction of their expression at 16 months; and, finally, (3) a visible reduction of miRNAs expression in obese mice at 4 months that remained until the end of the study. Based on previous pre-clinic studies, the miRNAs validated by RT-qPCR in our study are involved in alteration of glucose and lipid metabolism via insulin pathways (let-7f-5p, miR-10a-5p, miR-322)^20–22^, in cardiomyocytes apoptosis (miR-19b-3p, miR-25-3p, miR-30e-5p, miR-140-5p, miR-199a-3p, miR-499)^23–28^, in mitochondrial function (miR-181a/b)^29^, in pro-inflammatory signalling (miR-146a-5p, miR-155, miR-181b-3p, miR-3473b)^30–33^, and in cardiac hypertrophy (miR-451)^34^ and myocardial fibrosis process (miR-19b)^35,36^. Experimental details are described in Table 1 where we summarize a selection of studies whose results suggested a role of selected miRNAs in our animal model of diabetic cardiomyopathy.

Regarding the role of miR-19b in diabetic cardiomyopathy, Costantino and colleagues reported that miR-19b expression is up-regulated in myocardium of diabetic mice induced by streptozotocin administration (type I diabetes mellitus)^36^. On the contrary, we found miR-19b was downregulated in obese mice. Bugger and colleagues reported differences in the cardiac energetic metabolism between type 1 and type 2 diabetes animal models (induced by high-fat diet)^3,37^. We suggest that different sources of metabolic dysregulation may explain the observed differences in miR-19b expression levels between type 1 and type 2 diabetes animal models. On the other hand, miR-181b myocardial expression was not associated with cardiac dysfunction in diabetic cardiomyopathy animal models. However, recent works suggest that miR-181b may have a central role in vascular inflammation, inhibiting the activation of NF-κB signalling in endothelial cells^32^. Hori D and colleagues demonstrated that age-dependent decrease of miR-181b plays a critical role in vascular remodelling by activating TGF-β/pSmadD2/3 pathways^38^. In addition, miR-181b expression was markedly reduced in cardiac fibroblasts in response to Angiotensin II, suggesting its potential role in cardiac fibrosis^39^. Regarding insulin resistance mechanisms, Sun and colleagues reported that gene expression of miR-181b was decreased in adipose tissue and endothelial cells of obese mice, impairing glucose homeostasis, insulin sensitivity and promoting adipose tissue inflammation^40^.

Regarding circulating miRNAs as potential biomarkers of diabetic cardiomyopathy, we found an association between differential miRNA expressions in myocardium and plasma at 16 months in 8 miRNAs (let-7f-5p, miR-10a-5p, miR-19b-3p, miR-25-3p, miR-140-5p, miR-146a-5p, miR-181b-5p, miR-499-5p). To date, many clinical trials have proposed various circulating miRNAs as biomarkers for diabetes prognosis^41^. It was demonstrated that diabetic patients have a reduction of circulating let-7f-5p levels with respect to healthy individuals, but the concentration increases again with an anti-diabetic treatment^42^. Looking for pre-diabetes biomarkers, Nunez Lopez and colleagues identified a set of miRNAs, including miR-146a, that enable a classification to be made between lean, obese, and pre-diabetic patients^43^. Using a DM type 2 animal model (Zucker diabetic fatty rat), Delic and colleagues reported that circulating miR-140 decreases during the progression of diabetes^44^, propounding it as a potential biomarker for early manifestation of this disease. The biomarker properties of circulating miRNAs have been studied in cardiac diseases, in particular in the diagnosis of myocardial infarction^45^. Recently, Zhang and colleagues reported that plasma miR-499 increase is detectable as early as 1 h after onset of chest pain in acute myocardial infarction, proposing it as a sensitive biomarker for early diagnosis of a cardiac ischemic event^46^. These data coincide with previous results where it showed that miR-499 levels were increased in failing and hypertrophied human hearts, and in animal models of cardiac hypertrophy by miR-499 overexpression^47^. In our obese animal model, cardiac hypertrophy is present in spite of miR-499 being down regulated, showing that miR-208a/miR-499 pathway is not the driving force to generate the increase of heart mass and leaving open this possibility to other signal pathways like IGF-1, TGF-β or inflammatory cytokines related to diabetic cardiomyopathy (Table 1)^48,49^. The circulating miR-10a-5p was proposed as a non-invasive biomarker of transplant rejection in heart transplant recipients^50^, indicating worse prognosis and a reduction of its level in plasma. Fang and colleagues demonstrated that 16 miRNAs, including miR-19b serve as potential biomarkers for diffuse myocardial fibrosis in patients with hypertrophic cardiomyopathy with aortic stenosis and heart^51,52^. In addition, Karakas and colleagues showed that miR-19b could be a useful tool to predict cardiovascular mortality in patients diagnosed with stable angina pectoris or acute coronary syndrome in a prognostic study with a median follow-up of 4 years^53^. Seeger and colleagues reported that a decrease of miR-181b and miR-181c levels in serum correlate with changes in the course of immune-senescence accentuated in chronic heart failure patients, providing evidence for miR-181c as a biomarker of poor prognosis in patients with ischaemic or dilated cardiomyopathy^54^. Taking into account these previous studies about miR-19b and miR-181b as biomarkers in cardiac disease, we suggest the validation of their utilization as biomarkers for diabetic cardiomyopathy in a specific group of patients diagnosed with asymptomatic diabetes (related to obesity) without pre-existing coronary arterial disease in the context of primary prevention of cardiovascular disease.

In conclusion, we propose miR-19b and miR-181b as potential biomarkers for diabetic cardiomyopathy, with eventual application in clinical diagnosis to prevent metabolic and functional alteration in hearts of asymptomatic diabetic patients.

## Methods

### Animals

C57BL/6 male mice were housed at constant temperature (22 ± 2 °C) and humidity (60%), with a 12:12 hours light:dark cycle and unrestricted access to food and water.

Diabetic cardiomyopathy induction: All mice were fed with a regular diet up to and until five weeks of age. Then, they were kept on a regular diet (normal) or switched to high-fat diet (obese) until the end of the study (16 months of tested diet). Regular diet corresponded to 10 cal% fat, 20 cal% proteins and 70 cal% carbohydrates (Champion SA, Chile). High-fat diet corresponded to 60 cal% fat, 20 cal% proteins and 20 cal% carbohydrates (D12492, Research Diets Inc., USA). Animals were weighed weekly.

Animal experiments were performed in accordance with the Guide for Care and Use of Laboratory Animals^55^ and were approved by the Animal Care and Use Committee of the Faculty of Medicine, Clínica Alemana-Universidad del Desarrollo.

### Plasma glucose and NEFAs quantification

After six hours of fasting, blood samples were collected from the tail vein of alert mice. Plasma glucose levels were determined with the glucometer system Accu-Chek Performa (Roche Diagnostic, Germany). Plasma NEFA levels were measured using the Free Fatty Acid quantitation kit, (Sigma-Aldrich, USA).

### Glucose tolerance test

After six hours of fasting, mice received intraperitoneally 2 mg D-glucose/g body weight. Fifteen minutes before and 15, 30, 60, 90 and 120 minutes after D-glucose administration, blood glucose quantification was performed according with the method above described.

### Cardiovascular parameter assessment at basal and stress conditions

Mice were deeply anesthetized with 60 mg/Kg ketamine plus 4 mg/Kg xylazine and placed in supine position on a thermo-regulated plate. Body temperature was monitored using a rectal thermometer and gaseous oxygen was supplied. Hemodynamic parameters were measured by cardiac catheterization^56,57^. The catheter consisted of a Mikro-Tip SPR-671 pressure sensor (Millar, USA), which was coupled to the PCU-2000 transducer pressure/volt (Millar) and connected to the PowerLab 4/30 data acquisition system (AdInstruments, Australia). Hemodynamic parameters (arterial systolic and diastolic pressures, mean arterial pressure (MAP), Left ventricular end-diastolic pressure, (LVEDP), *d*P/*d*t_max_ and *d*P/*d*t_min_) were recorded under basal and stress conditions. For the latter, a PE-10 plastic tube (Warner Instruments Company, USA) was introduced into the mice jugular vein and connected to a KDS-KDS210P pump (Kdscientific Inc., USA) for dobutamine stimulation. The dobutamine infusion regime consisted of six, two-minute intervals, from 2 ng/g/min to 12 ng/g/min^58^. Data obtained were analysed with LabChart 7Pro software (AdInstruments, Australia).

### Cardiac macroscopic analysis

After the cardiac catheterization study, mice were euthanized with an overdose of ketamine/xylazine (60/4 mg/Kg) and hearts were removed. Heart samples were cryopreserved at −80 °C for miRNA analysis.

### Total RNA isolation

Frozen heart samples (50 mg approximately) were homogenised by pellet pestle (Sigma-Aldrich, USA). Blood was obtained by cardiac puncture and collected in a tube containing EDTA as anticoagulant. A volume of 300 μl of plasma was considered for each sample. Total RNA was extracted using miRVana PARIS (Applied Biosystems, USA) according to the manufacturer’s instructions. After the denaturation step, 0.6 fmol of Cel-miR-39 in 2 μl nuclease-free water was added. RNAs were eluted in free-nuclease-free water and frozen at −80 °C. RNA quality was assessed using the 2200 TapeStation System (Agilent Technologies, USA). Samples with a RIN (RNA integrity number) more than 7 were considered for further experiments.

### Microarray of microRNA

MiRNA expression patterns were examined by GeneChip miRNA 4.0 arrays (Affymetrix, USA), which was designed based on miRBase version 20. The array included 1908 probes for mature microRNAs and 1255 for pre-mature microRNAs. One microgram of total RNA was used as input into the labelling reaction as recommended by the protocol of the FlashTag Biotin HSR RNA Labelling Kit (Affymetrix, USA). Labelled miRNA was then hybridized to the array for 16 hours at 48 °C and 60 rpm. Then Genechips were scanned by using the GeneChip Scanner 3000 7 G (Affymetrix, USA).

### Microarray data analysis

CEL files were normalized using the RMA algorithm. Differentially expressed genes were identified using the t-test in TAC software (Affymetrix) by using an FDR value threshold of (<0.1) and an absolute fold-change > 2.

### Mapping miRNA to known pathways

In order to increase the power of the microarray analysis, the integrated information on validated miRNA-target interactions on KEGG pathways from miRWalk 2.0 was used. First, we used a less stringent criterion for selecting miRNAs for the microarray analysis. In this context, we used raw p-value of 0.05 as a threshold, selecting 94 miRNAs.

The MiRWalk2.0 database contains interactions of miRNA-targets of genes related to the specific process. KEGG pathways related to the experimental design were selected in order to obtain the interactions previously identified. The selected pathways were: “Apoptosis”, mmu04210; “Cardiac muscle contraction”, mmu04260; “Hypertrophic cardiomyopathy”, mmu05410; “Insulin signalling pathway”, mmu04910; and Type II diabetes mellitus, mmu04930. The miRNAs obtained from these KEGG pathways were matched with those from the list of selected miRNAs.

### RT-qPCR for microRNA expression

cDNA from isolated miRNA of myocardium and plasma were synthetized using the cDNA Taqman^®^ miRNA Reverse Transcription kit or TaqMan^®^ Advanced miRNA cDNA Synthesis Kit. Synthesized cDNA samples were then subjected to RT-qPCR using the TaqMan^®^ Universal PCR Master Mix II, No UNG or TaqMan^®^ Advanced miRNA Assays according to the manufacturer’s instructions (Applied Biosystems). The IDs of the probes are: 478139_mir, miR-499-5p; 465329_mat, miR-669m-5p; 001076, miR-322; 465212_mat, miR-3473b; 479241_mir, miR-10a-5p; 478583_mir, 181b-5p; 002571, miR-155-5p; 478399_mir, miR-146a-5p; 002562, miR-541; 001141, miR-451; 479235_mir, miR30e-5p; 478264_mir, miRNA-19b-3p; 478578_mir, let-7f-5p; 477909_mir, miR-140-5p; 477961_mir, miR-199a-3p; 477994_mir, miR-25-3p; 478293_mir, cel-miR-39-3p; 001973, U6 snRNA; 1232, snoRNA202 and 1234, snoRNA234.

Expression levels between normal and obese mice were quantitatively compared using the ΔΔCt method. The snoRNA202, snoRNA234 and U6 snRNA expression were analysis by NormFinder to choose the most suitable candidate to use it as an endogenous control in myocardium and cel-miR-39 as a spike-in miRNA for miRNA abundance normalization in plasma.

### Statistical analysis

Data presented as mean ± S.E.M. Normal distribution was tested by using the Kolmogorov-Smirnov test. To determine the statistical significances of intergroup differences, a two-way ANOVA test was used to compare mean values among all groups and Student’s unpaired *t*-test or Mann-Whitney test (non-parametric) was used to compare mean values between the two groups. Except for the microarray study, sample size as estimated considering a 5% of significance level and 80% of statistical power as described^59^. p < 0.05 was considered as statistically significant. GraphPad Prism version 4.03 software was used. For plasmatic miRNA levels comparison, we used a two ANOVA model followed with TukeyHSD post test in R software 3.4 version. MiRNA expression stability was determined using the NormFinder Visual Basic application for Microsoft Excel^60^.

## Electronic supplementary material

Supplementary materials

miR-19b and miR-181b gene targerts

miRNA Microarray Raw data
