# Supplementary material for: Circulating miR-19b and miR-181b are potential biomarkers for diabetic cardiomyopathy
Source: Sci Rep. 2017 Oct 18;7:13514. doi: 10.1038/s41598-017-13875-2 (PMC5647433; doi:10.1038/s41598-017-13875-2)
Supplement: Supplementary file 1 — Supplementary materials [file 41598_2017_13875_MOESM1_ESM.pdf]

Supplementary materials for

Circulating miR-19b and miR-181b are potential biomarkers for diabetic cardiomyopathy

Camila Uribe Copier[1#], Luis León[2#], Mauricio Fernández[3], David Contador[1] and Sebastián D. Calligaris[1*]

[#]Equal contribution

Camila Uribe Copier, David Contador and Sebastián D. Calligaris
[1]Centro de Medicina Regenerativa
Facultad de Medicina
Clínica Alemana Universidad del Desarrollo
Av. Las Condes 12.438, Lo Barnechea, Santiago, Chile

Luis León
[2]Instituto de Ciencias Biomédicas
Facultad de Ciencias de la Salud
Universidad Autónoma de Chile
Pedro de Valdivia 425, Providencia, Santiago, Chile

Mauricio Fernández
[3]Unidad de prevención cardiovascular
Clínica Alemana
Vitacura 5951, Vitacura, Santiago, Chile

Fig. 1S. Cardiovascular characteristics of the obesity animal model. (A) Mean arterial pressure (MAP) and (B) Left ventricular end-diastolic pressure (LVEDP) were measured at basal condition by cardiac catheterization; (C) Heart weight; and (D) Transversal heart sections were stained with haematoxylin/eosin. Mean cross-sectional area of cardiomyocytes (A[cmy]) was measured by a stereological method as described[1]. Solid and dotted lines indicate normal and obese mice respectively: (n= 8, per A, B, C plots and n=3, per D plot). Mean ± SEM, *: $p<0.05$, **: $p<0.01$ vs. normal mice (Two-way ANOVA test).

Fig. 2S. Correlation study. Physiological parameters were correlated with miRNAs expression levels using Spearman correlations, which were visualized as a matrix using the corrplot package in R. "N_" and "O_" means Normal and Obese respectively. n=8

Table 1S. Results of miRNA microarray from myocardium of obese and normal mice at 16 months.

References:

1 Mandarim-de-Lacerda, C. A. Stereological tools in biomedical research. *An Acad Bras Cienc* **75**, 469-486 (2003).
